# Risk of epilepsy in stroke patients receiving acupuncture treatment: a nationwide retrospective matched-cohort study

**DOI:** 10.1136/bmjopen-2015-010539

**Published:** 2016-07-13

**Authors:** Shu-Wen Weng, Chien-Chang Liao, Chun-Chieh Yeh, Ta-Liang Chen, Hsin-Long Lane, Jaung-Geng Lin, Chun-Chuan Shih

**Affiliations:** 1 Graduate Institute of Chinese Medicine, College of Chinese Medicine, China Medical University, Taichung, Taiwan; 2 Department of Chinese Medicine, Taichung Hospital, Ministry of Health and Welfare, Taichung, Taiwan; 3 Department of Anesthesiology, Taipei Medical University Hospital, Taipei, Taiwan; 4 Department of Anesthesiology, School of Medicine, College of Medicine, Taipei Medical University, Taipei, Taiwan; 5 Health Policy Research Center, Taipei Medical University Hospital, Taipei, Taiwan; 6 Department of Surgery, China Medical University Hospital, Taichung, Taiwan; 7 Department of Surgery, University of Illinois, Chicago, Illinois, USA; 8 School of Chinese Medicine for Post-Baccalaureate, I-Shou University, Kaohsiung City, Taiwan; 9 Department of Healthcare Administration, Asia University, Taichung, Taiwan; 10 Program for the Clinical Drug Discovery from Botanical Herbs, College of Pharmacy, Taipei Medical University, Taipei, Taiwan

**Keywords:** Acupuncture

## Abstract

**Objective:**

To investigate the risk of epilepsy in stroke patients receiving and not receiving acupuncture treatment.

**Design:**

Retrospective cohort study.

**Setting:**

This study was based on Taiwan's National Health Insurance Research Database that included information on stroke patients hospitalised between 1 January 2000 and 31 December 2004.

**Participants:**

We identified 42 040 patients hospitalised with newly diagnosed stroke who were aged 20 years and above.

**Primary and secondary outcome measures:**

We compared incident epilepsy during the follow-up period until the end of 2009 in stroke patients who were and were not receiving acupuncture. The adjusted HRs and 95% CIs of epilepsy associated with acupuncture were calculated using multivariate Cox proportional hazard regression.

**Results:**

Stroke patients who received acupuncture treatment (9.8 per 1000 person-years) experienced a reduced incidence of epilepsy compared to those who did not receive acupuncture treatment (11.5 per 1000 person-years), with an HR of 0.74 (95% CI 0.68 to 0.80) after adjustment for sociodemographic factors and coexisting medical conditions. Acupuncture treatment was associated with a decreased risk of epilepsy, particularly among stroke patients aged 20–69 years. The log-rank test probability curve indicated that stroke patients receiving acupuncture treatment had a reduced probability of epilepsy compared with individuals who did not receive acupuncture treatment during the follow-up period (p<0.0001).

**Conclusions:**

Stroke patients who received acupuncture treatment had a reduced risk of epilepsy compared with those not receiving acupuncture treatment. However, the protective effects associated with acupuncture treatment require further validation in prospective cohort studies.

**Strengths and limitations of this study**

- We used propensity score matching to reduce confounding effects in this study, and multivariate Cox proportional hazard models were used to control the residual confounding bias.
- Our study had no selection bias because all stroke patients hospitalised from 2000 to 2004 in Taiwan were analysed.
- Our data lack information on clinical risk scores, lesion characteristics, biochemical measures and the lifestyles of stroke patients,  and preventive administration of antiepileptic medications or engagement with rehabilitation programmes during the follow-up period were not considered.
- The lack of validity of diagnostic disease codes is also a limitation of the study; in addition our study could not validate the actual acupuncture points used in treatment.
- The mode of acupuncture treatment for stroke patients varied among traditional Chinese medicine (TCM) physicians.

## Introduction

Stroke remains the leading cause of adult disability and death worldwide.^1^ ^2^ Approximately 5.8 million deaths globally were attributed to stoke in 2010 and the annual cost of stroke was estimated to be approximately US$30 000 per patient worldwide.^2^ ^3^ Given that strokes occur in 2.8% of adults in the USA, the economic burden of stroke in the USA was US$36.5 billion in 2010.^4^ In Taiwan, the average medical costs in the first year after stroke were US$5679 per patient in 1997–2002.^5^ Epilepsy, depression, headache, acute myocardial infarction, sleep disorders, pneumonia, gastrointestinal bleeding, urinary tract infection, musculoskeletal pain, recurrent stroke and post-stroke falls are common medical complications after stroke.^6–8^

Acupuncture is a major part of traditional Chinese medicine (TCM) and is widely used in many countries.^9^ A previous study found that TCM is commonly employed for stroke patients in Asian countries,^10^ and several investigations have demonstrated that acupuncture treatment may improve dysphagia,^11^ shoulder pain,^12^ spasticity,^13^ disability and quality of life in stroke patients.^14^ ^15^ Epilepsy frequently occurs after patients experience haemorrhagic stroke, with an estimated prevalence ranging from 4.1% to 11.3%.^16^ ^17^ After the first seizure, patients may develop recurrent seizures and exhibit increased fatality.^17^ ^18^ Although acupuncture treatment is helpful and potentially reduces seizure frequency in epilepsy patients,^19^ limited information is available regarding whether acupuncture treatment is beneficial in reducing epilepsy in stroke patients.

Using the Taiwan National Health Insurance Research Database (NHIRD), we conducted a nationwide, propensity score-matched, population-based, retrospective cohort study to investigate the risk of epilepsy in stroke patients who did and did not receive acupuncture treatment.

## Methods

### Source of data

Research data were obtained from the reimbursement claims of the Taiwan National Health Insurance Program, which was implemented in March 1995 and insures over 99% of the 23 million residents in Taiwan. NHIRD was established by the National Health Research Institutes in Taiwan and records all beneficiaries’ medical services, inpatient and outpatient demographics, primary and secondary diagnoses, procedures, prescriptions and medical expenditures, in the interest of public research. The validity of this database has been favourably evaluated, and research articles that have used this database have been published in prominent scientific journals worldwide.^10^ ^20–25^

### Ethical statements

The insurance reimbursement claims used in this study were from Taiwan's NHIRD. To ensure privacy, the patients were de-identified in the electronic database. This study was evaluated and approved by the Institutional Review Board of Taiwan's National Health Research Institutes (NHIRD-100-122) and E-DA Hospital, Kaohsiung, Taiwan (2014012). Informed consent was not required because the patients’ identities were decoded and scrambled. This study was conducted in accordance with the Declaration of Helsinki.^10^ ^20–25^

### Study design

In this study, we used a similar approach using the same database in previous works.^24^ ^25^ We identified patients ≥20 years of age with newly diagnosed stroke who were hospitalised between 2000 and 2004 as eligible study subjects. Each subject either received follow-up from the index date until 31 December 2009 or was censored. To confirm that all stroke patients in our study were incident cases, only new-onset stroke cases were included; patients with a diagnosis of stroke within a 4-year period prior to the study were not included. Overall, we identified 226 699 new-onset stroke survivors ≥20 years of age among the 23 million people in Taiwan; 21 020 of these patients had received at least two courses (one course consisted of six consecutive treatments) of acupuncture treatment after being discharged. We compared stroke patients receiving at least two courses of acupuncture treatment with patients not receiving acupuncture treatment. We randomly selected stroke patients who had not received acupuncture treatment as controls (case–control ratio 1:1); these patients were matched according to age, sex, stroke subtype, low income, urbanisation, hypertension, mental disorder, diabetes, hyperlipidaemia, head injury, Parkinson's disease, Alzheimer's disease, brain cancer, renal dialysis, rehabilitation, anti-epilepsy drugs, anticoagulant, anti-platelet agents, lipid-lowering agents, stay in intensive care unit (ICU), neurosurgery and length of hospital stay.

### Criteria and definition

We defined stroke according to the International Classification of Diseases, 9th Revision, Clinical Modification (ICD-9-CM 430–438). Eligible study participants included the 226 699 patients in the stroke cohort. Coexisting medical conditions included hypertension (ICD-9-CM 401–405), mental disorder (ICD-9-CM 290–319), diabetes (ICD-9-CM 250), hyperlipidaemia (ICD-9-CM 272.0–272.4), head injury (ICD-9-CM 800–804, 850–854), Parkinson's disease (ICD-9-CM 332), Alzheimer's disease (ICD-9-CM 331), brain cancer (ICD-9-CM 191) and regular renal dialysis (administration codes D8 and D9). Use of rehabilitation, anti-epilepsy drugs, anticoagulant, anti-platelet agents and lipid-lowering agents during the follow-up period was also considered in this study. During the index stroke admission, we identified stay in ICU, neurosurgery and length of hospital stay as potential confounding factors for the association between acupuncture treatment and post-stroke epilepsy. Under the condition of not experiencing previous epilepsy prior to stroke admission before 1 January 1996, cases of newly diagnosed epilepsy (ICD-9-CM 345) were further defined as at least one medical visit (including inpatient and outpatient care) with a physician's diagnosis after stroke admission.

### Statistical analysis

We used a propensity score-matched pair method combined with frequency matching to analyse patients with and without acupuncture treatment. We developed a non-parsimonious multivariable logistic regression model to estimate a propensity score for patients with and without acupuncture treatment, irrespective of outcome. Clinical significance guided the initial choice of covariates in this model: age, sex, stroke subtype, low income, urbanisation, hypertension, mental disorder, diabetes, hyperlipidaemia, head injury, Parkinson's disease, Alzheimer's disease, brain cancer, renal dialysis, rehabilitation, anti-epilepsy drugs, anticoagulant, anti-platelet agents, lipid-lowering agents, stay in ICU, neurosurgery and length of hospital stay. We matched patients with acupuncture to patients without acupuncture using a greedy-matching algorithm with best to next-best matches until no more matches could be made. In the 1:1 matching procedure, all cases are initially matched to their ‘best’ control in the first iteration of the 8 to 1 Digit Match. The set of matched cases is then matched to the set of unmatched controls in N–1 additional iterations of the 8 to 1 Digit Match. If a case does not have one matched control, it is removed from the set of matches at the time it fails to receive a matched control. The corresponding control is also removed from the set of matches. The control is added back to the pool of unmatched controls, and allowed to match another case. This method can remove 98% of the bias from measured covariates. The χ^2^ test was used to measure covariate balance and a p value <0.05 was suggested to represent meaningful covariate imbalance.

The follow-up time, in person-years, was calculated for each subject until the diagnosis of epilepsy or until censoring because of death, withdrawal from the insurance system, or loss to follow-up. The outcome of this study was the person-years of new-onset epilepsy in stroke patients. This study's objective was to determine whether using acupuncture was associated with a reduced incidence of epilepsy in stroke patients.

We first compared the distribution of sociodemographic factors and coexisting medical conditions between the stroke cohorts with and without acupuncture treatment using χ^2^ tests and t tests. We calculated HRs with 95% CIs for the risk of epilepsy after stroke associated with acupuncture treatment, adjusting for age, sex, low income and urbanisation in multivariate Cox proportional hazard regression models. The duration of observation for each person was calculated as the time until the individual was diagnosed with epilepsy or censored for death, migration, or discontinued enrolment in the insurance system. Adjusted HRs with 95% CIs for epilepsy associated with acupuncture treatment were calculated using multivariate Cox proportional hazard analyses with the variables categorised. We also performed age- and sex-stratified analyses to investigate the association between epilepsy and the use of acupuncture. SAS software V.9.1 (SAS Institute, Cary, North Carolina, USA) was used for data analyses with a two-tailed probability, and p<0.05 was considered statistically significant.

## Results

After the propensity score matching procedure, no significant differences in sex, age, stroke subtype, low income, urbanisation, coexisting medical condition, ICU stay, neurosurgery or length of hospital stay were noted between stroke patients with and without acupuncture treatment (table 1).

**Table 1:** Baseline characteristics of stroke patients with and without acupuncture treatment

	Acupuncture use		
	No (N=21 020)	Yes (N=21 020)	
	n (%)	n (%)	p Value
Female	9272 (44.1)	9272 (44.1)	1.0000
Age, years			1.0000
20–29	75 (0.4)	75 (0.4)	
30–39	284 (1.4)	284 (1.4)	
40–49	1878 (8.9)	1878 (8.9)	
50–59	4320 (20.6)	4320 (20.6)	
60–69	7080 (33.7)	7080 (33.7)	
70–79	6311 (30.0)	6311 (30.0)	
≥80	1072 (5.1)	1072 (5.1)	
Stroke subtype			1.0000
Haemorrhage	1775 (8.4)	1775 (8.4)	
Ischaemic	12 285 (58.4)	12 285 (58.4)	
Other	6960 (33.1)	6960 (33.1)	
Low income	184 (0.9)	184 (0.9)	1.0000
Urbanisation			1.0000
Low	582 (2.8)	582 (2.8)	
Moderate	7247 (34.5)	7247 (34.5)	
High	13 191 (62.8)	13 191 (62.8)	
Coexisting medical condition			
Hypertension	15 887 (75.6)	15 887 (75.6)	1.0000
Mental disorder	8144 (38.7)	8144 (38.7)	1.0000
Diabetes mellitus	8048 (38.3)	8048 (38.3)	1.0000
Hyperlipidaemia	3403 (16.2)	3403 (16.2)	1.0000
Head injury	1659 (7.9)	1659 (7.9)	1.0000
Parkinson's disease	656 (3.1)	656 (3.1)	1.0000
Alzheimer's disease	13 (0.1)	13 (0.1)	1.0000
Brain cancer	4 (0.0)	4 (0.0)	1.0000
Renal dialysis	304 (1.5)	304 (1.5)	1.0000
Rehabilitation	13 803 (46.6)	13 803 (46.6)	1.0000
Anti-epilepsy drugs	6170 (29.4)	6170 (29.4)	1.0000
Anticoagulant	1599 (7.6)	1599 (7.6)	1.0000
Anti-platelet agents	20 016 (95.2)	20 016 (95.2)	1.0000
Lipid-lowering agents	11 307 (53.8)	11 307 (53.8)	1.0000
ICU stay	1412 (6.7)	1412 (6.7)	1.0000
Neurosurgery	318 (1.5)	318 (1.5)	1.0000
Length of hospital stay, days	6.92±5.19	6.93±5.25	0.7302

ICU, intensive care unit.

Compared to stroke patients who did not receive acupuncture treatment (table 2), stroke patients receiving acupuncture treatment exhibited a reduced incidence of epilepsy (9.8 vs 11.5 per 1000 person-years, p<0.0001). After adjustment for sex, age, stroke subtype, low income, urbanisation, coexisting medical condition, rehabilitation, anti-epilepsy drugs, anticoagulant, anti-platelet agents, lipid-lowering agents, ICU stay, neurosurgery and length of hospital stay, stroke patients receiving acupuncture treatment exhibited a decreased risk of epilepsy (HR 0.74; 95% CI 0.68 to 0.80) compared with those not receiving acupuncture treatment. The association between acupuncture treatment and reduced risk of post-stroke epilepsy was significant in men (HR 0.77; 95% CI 0.69 to 0.85) and women (HR 0.70; 95% CI 0.61 to 0.81), stroke patients aged 20–69 years as well as in individuals who had experienced haemorrhagic stroke (HR 0.60; 95% CI 0.50 to 0.73), ischaemic stroke (HR 0.86; 95% CI 0.78 to 0.96) and other stroke (HR 0.62; 95% CI 0.52 to 0.74). The HR values of post-stroke epilepsy were 0.16 (95% CI 0.04 to 0.68), 0.39 (95% CI 0.21 to 0.73), 0.51 (95% CI 0.39 to 0.66), 0.66 (95% CI 0.54 to 0.80) and 0.79 (95% CI 0.68 to 0.91) in stroke patients aged 20–29 years, 30–39 years, 40–49 years, 50–59 years and 60–69 years, respectively.

**Table 2:** Incidence and risk of epilepsy in stroke patients with and without acupuncture treatment by age, sex and stroke type

	No acupuncture				Acupuncture use					
	n	Events	Person-years	Incidence	n	Events	Person-years	Incidence	HR	(95% CI)
All*	21 020	1382	120 164	11.5	21 020	993	101 044	9.8	0.74	(0.68 to 0.80)
Female†	9272	525	55 162	9.5	9272	343	45 620	7.5	0.70	(0.61 to 0.81)
Male†	11 748	857	65 001	13.2	11 748	650	55 424	11.7	0.77	(0.69 to 0.85)
Age, years‡										
20–29	75	9	408	22.1	75	3	369	8.1	0.16	(0.04 to 0.68)
30–39	284	31	1632	19.0	284	16	1363	11.7	0.39	(0.21 to 0.73)
40–49	1878	165	11 601	14.2	1878	90	9357	9.6	0.51	(0.39 to 0.66)
50–59	4320	263	26 875	9.8	4320	166	22 064	7.5	0.66	(0.54 to 0.80)
60–69	7080	444	42 234	10.5	7080	332	34 762	9.6	0.79	(0.68 to 0.91)
70–79	6311	403	33 073	12.2	6311	341	29 006	11.8	0.88	(0.76 to 1.02)
≥80	1072	67	4340	15.4	1072	45	4123	10.9	0.71	(0.48 to 1.04)
Type of stroke§										
Haemorrhage	1775	253	9355	27.0	1775	179	8501	21.1	0.60	(0.50 to 0.73)
Ischaemic	12 285	764	68 961	11.1	12 285	615	59 265	10.4	0.86	(0.78 to 0.96)
Other	6960	365	41 848	8.7	6960	199	33 277	6.0	0.62	(0.52 to 0.74)

*Adjusted for all covariates in table 1.

†Adjusted for covariates in table 1 except sex.

‡Adjusted for covariates in table 1 except age.

§Adjusted for covariates in table 1 except stroke subtype.

**Figure 1:**
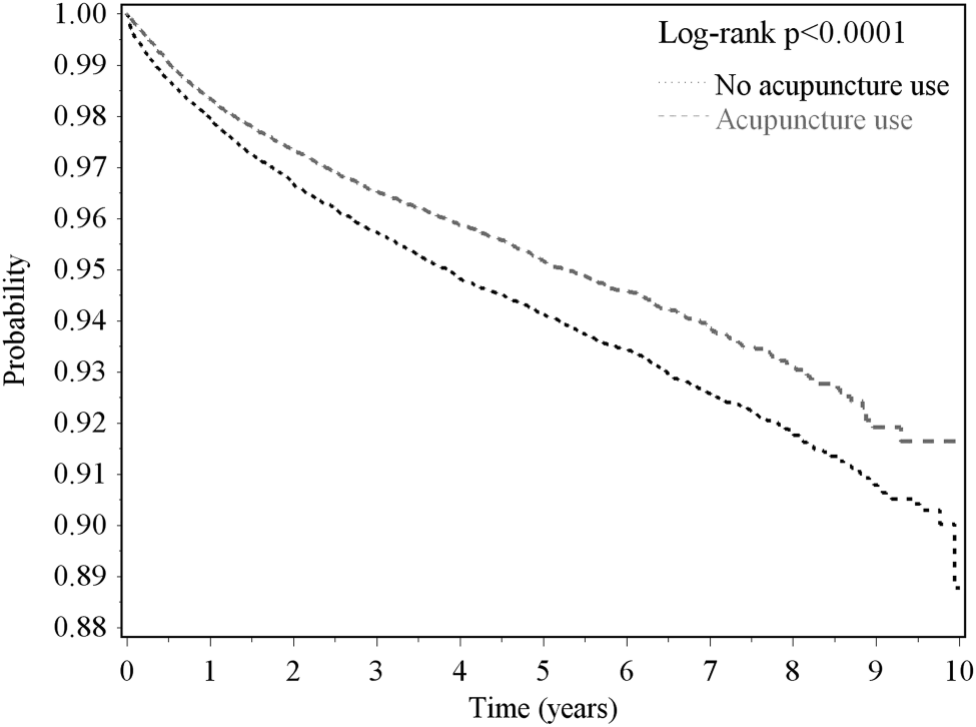
Probability of no epilepsy in post-stroke patients with and without acupuncture treatment estimated using the Kaplan-Meier method.

Among stroke patients, the risk of epilepsy exhibited a dose-dependent decrease with increasing use of acupuncture treatment (table 3). Figure 1 shows that the probability of epilepsy in patients receiving acupuncture was reduced compared to that in patients not receiving acupuncture.

**Table 3:** Risk of epilepsy and number of acupuncture courses in stroke patients

Number of courses	n	Events	Person-years	Incidence	HR (95% CI)*
0	21 020	1382	120 164	11.5	0.77 (0.66 to 0.90)
2	4385	182	18 258	10.0	1.06 (0.91 to 1.25)
3	2907	164	12 720	12.9	0.84 (0.69 to 1.02)
4	2040	104	9597	10.8	0.75 (0.59 to 0.95)
5	1545	72	7271	9.9	0.74 (0.55 to 0.98)
6	1168	48	5464	8.8	0.64 (0.57 to 0.71)
≥7	8975	423	47 734	8.9	0.69 (0.63 to 0.74)

*Adjusted for age, sex, stroke subtype, low income, urbanisation, hypertension, mental disorder, diabetes, hyperlipidaemia, head injury, Parkinson's disease, Alzheimer's disease, brain cancer, renal dialysis, rehabilitation, anti-epilepsy drugs, anticoagulant, anti-platelet agents, lipid-lowering agents, stay of intensive care unit, neurosurgery and length of hospital stay.

## Discussion

In this nationwide, propensity score-matched, retrospective cohort study, we observed that stroke patients receiving acupuncture treatment exhibited a significantly lower risk of epilepsy during the follow-up period compared to those not receiving acupuncture treatment. A decreased risk of epilepsy after receiving acupuncture treatment was noted only among stroke patients aged 20–69 years. To our knowledge, this study is the first to report an association between using acupuncture and a reduced risk of post-stroke epilepsy.

Older age, male sex, urbanisation, hypertension, diabetes mellitus, mental disorder, Parkinson's disease and Alzheimer's disease are known risk factors associated with stroke.^26^ ^27^ These sociodemographic factors and coexisting medical conditions are also associated with epilepsy.^6–23^ ^26–30^ Neurosurgery, ICU stay and length of hospital stay have also been shown to have an impact on post-stroke epilepsy.^7^ ^31^ To reduce bias, we used propensity score matching and multivariate regression to control for potential confounders.

Our study demonstrated that acupuncture treatment is associated with a decreased risk of epilepsy among male and female stroke patients aged 20–69 years. Age and sex have been previously shown to determine the medical conditions and complications of individuals with stroke.^20^ For instance, older populations exhibit an increased mortality rate and poorer outcomes after stroke.^32^ Young and middle-aged adults have fewer coexisting medical conditions, have better medical knowledge, better attitudes towards health and healthier lifestyles, and more commonly use TCM or acupuncture treatment compared with older individuals.^10^ ^33^ These findings may partly explain why we observed enhanced effects of acupuncture treatment which decreased the risk of post-stroke epilepsy in young and middle-aged adults.

The incidence of post-stroke epilepsy varied depending on stroke type.^16^ ^34^ Consistent with previous reports,^16^ ^17^ the present study also demonstrated that patients who experienced haemorrhagic stroke exhibited an increased risk of developing seizures compared to those who experienced ischaemic stroke or other stroke subtypes. Nevertheless, our investigation indicated the beneficial effects of acupuncture treatment in reducing the risk of epilepsy in patients with every stroke subtype.

Acupuncture treatment is one option for patients undertaking rehabilitation programmes,^35^ particularly stroke patients.^36^ We propose three possible explanations for the association between acupuncture treatment and the decreased risk of post-stroke epilepsy that was demonstrated in this study. First, acupuncture intervention stimulates multiple levels of differential activity over a wide range of brain networks and this modulation and sympathovagal response may be related to acupuncture analgesia and other potential therapeutic effects.^37^ Second, depression is a frequent coexisting condition in stroke patients. Indeed, a meta-analysis reported that acupuncture shows a beneficial effect on patients with depression.^38^ Stroke patients with coexisting medical conditions, such as dementia or Alzheimer's disease, are also more likely to develop epilepsy,^39^ and an interventional trial reported that acupuncture treatment potentially improves cognitive function and quality of life in vascular dementia patients.^40^ Third, stroke patients who receive acupuncture treatment may have more knowledge about and better attitudes towards physical rehabilitation, which is helpful in reducing post-stroke epilepsy, compared to patients not receiving acupuncture treatment.

Functional MRI used in previous studies indicated that laser acupuncture activated the precuneus which is relevant to mood in the posterior default mode network, while needle acupuncture activated the parietal cortical region associated with the primary motor cortex.^41^ In addition, laser stimulation of acupoints led to activation of frontal-limbic-striatal brain regions, with the pattern of neural activity somewhat different for each acupuncture point.^42^ Acupuncture regulates functional connectivity by activation in under-activated brain regions, deactivation where there is overload and over-activation and so on. This may be one of the reasons why there is a reduction in epilepsy. Therefore, we believe that laser acupuncture may also be helpful during rehabilitation in stroke patients. In addition, the neurological effects of acupuncture was investigated in our previous study that reported the decreased risk of stroke in patients with traumatic brain injury receiving acupuncture treatment.^24^ We also reported that stroke patients who received acupuncture treatment may have decreased risk of acute myocardial infarction previously.^25^ Therefore, it may be reasonable that reduced post-stroke epilepsy was investigated in stroke patients receiving acupuncture treatment.

One of the strengths of this study was its large sample size and reduced selection bias. Second, our study consisted of a retrospective cohort design which used the NHIRD; this design provides stronger causal inference than case–control or cross-sectional designs. Third, to eliminate the influence of sociodemographic factors and coexisting medical conditions between stroke patients receiving and not receiving acupuncture treatment, we used a propensity score matched-pair procedure to select acupuncture and non-acupuncture treatment controls. To control for residual confounding effects in the association between a decreased risk of post-stroke epilepsy and acupuncture treatment, we applied multivariable Cox proportional hazard models to calculate the adjusted HRs and 95% CIs of epilepsy associated with acupuncture treatment. Finally, immortal time in observational studies may bias the results in favour of the treatment group, thereby overestimating the beneficial effects. To reduce such bias, we calculated person-years by correcting for immortal time in the acupuncture treatment group.

Nevertheless, this study had certain limitations. First, we used insurance claims data, which lack information on clinical risk scores (such as the National Institute of Health Stroke Scale or the Barthel Index), lesion characteristics (location or size), biochemical measures and patients’ lifestyles, which have been reported as predictors of post-stroke epilepsy. Thus, this study does not provide information regarding the severity-dependent relationship between epilepsy and stroke. Second, preventive administration of antiepileptic medications or other rehabilitation programmes were not considered during the follow-up period. Thus, we were unable to evaluate the influence of these factors on the association between acupuncture and the decreased risk of post-stroke epilepsy. Third, although the accuracy of diagnosis codes in the database has been validated in previous studies,^10^ the validity of co-morbidity codes is one potential limitation of the study. The prevalence of epilepsy may also have been underestimated, given that patients experiencing mild symptoms may not seek medical treatment; however, these effects appear minimal in our population-based study. In addition, our study could not validate the actual acupuncture points used in treatment, given the limited information provided by the reimbursement claims in Taiwan's NHIRD. Finally, the mode of acupuncture treatment for stroke patients varied among TCM physicians. Thus, we could not confirm that all TCM physicians performed the same procedures and used the same acupuncture points for stroke patients.

In conclusion, stroke patients receiving acupuncture treatment exhibited a reduced risk of epilepsy compared to patients not receiving acupuncture treatment. The association between decreased epilepsy risk and acupuncture use was observed in men, women, younger adults, and patients with any stroke subtype. However, this associated protective effect must be further assessed in randomised clinical trials to provide direct evidence regarding the effectiveness and mechanisms of acupuncture treatment for post-stroke epilepsy.
